# Amylase-Producing Maltooligosaccharide Provides Potential Relief in Rats with Loperamide-Induced Constipation

**DOI:** 10.1155/2020/5470268

**Published:** 2020-08-27

**Authors:** Eun Yeong Jang, Yejin Ahn, Hyung Joo Suh, Ki-Bae Hong, Kyungae Jo

**Affiliations:** ^1^Department of Integrated Biomedical and Life Science, Graduate School, Korea University, Seoul 02841, Republic of Korea; ^2^Department of Public Health Science, Graduate School, Korea University, Seoul 02841, Republic of Korea; ^3^BK21 Plus, College of Health Science, Korea University, Seoul 02841, Republic of Korea

## Abstract

Constipation is a chronic disease caused by infrequent, inadequate, and difficult bowel movements. The present study aimed to evaluate the potential laxative effect of maltooligosaccharide (MOS) on loperamide-induced constipation in a rat model. *In vitro* experiments were conducted to evaluate the effect of MOS on the growth of lactic acid bacteria. Moreover, to examine the effect of MOS administration on Sprague-Dawley (SD) rats with loperamide-induced constipation, the drinking water for the rats was supplemented with 10% or 15% of MOS for 14 days, and, thereafter, the improvement in constipation was assessed. For this, the rats were divided into five groups: normal (Nor), loperamide-induced constipated (Con), positive control (15% of dual-oligosaccharide (DuO-15)), 10% MOS treated (MOS-10), and 15% MOS-treated (MOS-15). In an *in vitro* test, MOS treatment promoted the growth of lactic acid bacteria except *Lactobacillus bulgaricus*. Treatment with higher MOS dose relieved constipation in rats by improving the fecal pellet and water content. Furthermore, in the high MOS dose group, the cecal short-chain fatty acid levels significantly increased compared to those in the control group (*P* < 0.001). MOS treatment also improved the mucosal thickness as well as mucin secretion and increased the area of intestinal Cajal cells compared to that in the control group (*P* < 0.001). These findings suggest that MOS relieves constipation and has beneficial effect on the gastrointestinal tract, and, therefore, it can be used as an ingredient in functional foods for treating constipation or improving intestinal health.

## 1. Introduction

As people have become more concerned about their health, consumption of healthier foods has increased, thereby reducing disease risk. In this sense, certain types of dietary carbohydrates, particularly functional oligosaccharides, have gained immense interest. Oligosaccharides are low-molecular carbohydrates comprising 3–10 monosaccharides, such as glucose, fructose, and galactose [1]. Functional oligosaccharides such as galactooligosaccharide (GOS), fructooligosaccharide (FOS), and maltooligosaccharide (MOS) are well-known prebiotics owing to their ability to selectively stimulate beneficial bacteria in the intestines, particularly bifidobacterial species [2, 3]. These oligosaccharides increase the production of short-chain fatty acids by bifidobacteria and further stimulate intestinal peristalsis and increase fecal water content through osmotic pressure [4]. The most abundant sources of functional oligosaccharides are seaweeds and plants.

Starch, a carbohydrate stored in higher plants, is a homopolysaccharide comprising two types of glucose polymers, amylose and amylopectin. Amylose is an unbranched homopolysaccharide with *α*-(1 ⟶ 4) glycosidic linkages, whereas amylopectin is a branched homopolysaccharide with *α*-(1 ⟶ 4) and *α*-(1 ⟶ 6) glycosidic linkages. Starch acts as a substrate for producing oligosaccharides. MOS, produced by *α*-amylase (EC 3.2.1.1) and pullulanase (EC 3.2.1.9), is a mixture of linear oligosaccharides comprising two (G2), three (G3), four (G4), five (G5), or six (G6) glucose units joined by *α*-(1 ⟶ 4) glycosidic linkages [5]. MOSs serve as digestive nutritional sweeteners with low calorie content and osmotic pressure and are widely used in processing functional foods [6]. They display physiological characteristics that relieve fatigue, improve visceral peristalsis, and prevent constipation [3].

The MOS used in this study was prepared using only *α*-amylase without pullulanase. MOS containing undigested amylopectin has prebiotic activity that selectively stimulates the growth and function of the intestinal microflora. This type of alpha-amylase-resistant starch has prebiotic properties similar to those of resistant starch. Microflora changes resulting from the MOS-supplemented diet are beneficial to the digestive tract. Previous studies on constipation focused on fiber and nondigested oligosaccharides [7, 8]. In particular, isomaltooligosaccharide (IMOS) produced via hydrolysis and transglycosylation from soluble starch has been used to treat chronic constipation, and Goulas et al. [9] reported that at least 8 g/day of IMOS would be required to substantially elevate the bifidobacterial cell number in the human gut. Moreover, administration of 90% IMOS at a concentration of 8 g/day/kg of body weight in mice with loperamide-induced constipation increased the water content of the stool, shortened intestinal transit time, and increased stool short-chain fatty acid (SCFA) concentration [10]. Similar to MOS, IMOS is prepared using starch as the raw material; however, the process is complex as it requires a combination of enzymes immobilized in a two-stage reactor. Previous studies demonstrated that the laxative effect of MOS was similar to IMOS; however, the difference was not significant. Additionally, no further studies confirmed the effect of MOS is low doses. Therefore, in the present study, we evaluated the laxative potential of MOS with *α*-amylase-resistant starch prepared from organic rice.

## 2. Materials and Methods

### 2.1. Experimental Animals and Reagents

The Institutional Animal Care and Use Committee of Korea University (KU-IACUC; Approval Number KU-2019-0012) approved the use of animals for this study. The animals used in the experiment were Sprague-Dawley (SD) male rats (weighing 160–180 g) at 6 weeks of age (OrientBio; Seongnam, Korea). After adapting to the environment for 7 days, the experimental animals were randomly divided into 5 groups of 8 rats each, and each rat was bred for 25 days in an individual cage. During the experimental period, water and food were provided *ad libitum* at a room temperature of 20–22°C and a relative humidity of 50%–55% under a 12 h light/dark cycle. The MOS and dual-oligosaccharide (DuO) were supplied by NEO CREMAR Co. Ltd. (Seoul, Republic of Korea). Loperamide (L4762) was purchased from Sigma-Aldrich (St. Louis, MO, USA).

### 2.2. Bacterial Strains and Growth Conditions

*Lactobacillus fermentum* KCTC 3112*, L. paracasei* KCTC 3510*, L. reuteri* KCTC 3594*, L. bulgaricus* KCTC 3536*, Bifidobacterium breve* KCTC 3220, and *B. lactis* KCTC 5854 were purchased from the Korean Collection for Type Cultures (Daejeon, Republic of Korea) for evaluating the effect of MOS on the proliferation of lactic acid bacteria. Lactic acid bacteria were inoculated into modified peptone yeast extract fructose medium (PYF) containing 1%, 2%, or 4% of MOS and incubated at 37°C for 48 h. To determine the bacterial growth rate, culture samples were collected at 12 h intervals and absorbance was measured at 660 nm.

### 2.3. Determination of MOS Constipation Mitigation Effect

After one week of adaptation, constipation was induced by intraperitoneal administration of loperamide (3 mg/kg) once a day for 6 days in the experimental groups except for the untreated control group (Nor). The mice with constipation were divided into negative control (Con), positive control (DuO-15), low-dose MOS administration (MOS-10), and high-dose MOS administration (MOS-15) groups. The Nor and Con groups were supplied with only drinking water during the 2-week recovery period, whereas the DuO, MOS-10, and MOS-15 groups were supplied with drinking water supplemented with 15% of DuO, 10% of MOS, and 15% of MOS, respectively. Rats were sacrificed using carbon dioxide (CO_2_), and intestinal and cecum were collected for further analysis.

### 2.4. Measurement of Fecal Parameters

Fecal samples were collected thrice a week at 10 am during the sample processing period. The number of fecal pellets and wet fecal weight were measured. Fecal water content was calculated by drying the stool in an oven at 60°C for 24 h, measuring the dry weight, and calculating the difference between the wet and dry fecal weights.

### 2.5. Intestinal Transit Ratio

Intestinal transit ratio of MOS was measured using a modified method based on that of Kim et al. [11]. To investigate the effect of MOS on dietary transport, after 2 weeks of sample treatment, 1 ml of 8% (W/V) activated carbon was orally administered to the rats, and after 30 min, the gastrointestinal tract was extracted. The length of the intestine was calculated by making an incision after measuring the lengths of the small and large intestine. The intestinal transit ratio was calculated by dividing the total intestinal distance by the distance traveled by activated charcoal.

### 2.6. Determination of SCFAs in Feces

To determine the SCFA content, 1 g of cecal contents was collected 1 day before the end of the experiment, followed by SCFA extraction using 5 ml of methanol and filtering via a 0.45 *μ*m Millipore filter (Millipore, USA). SCFAs were analyzed using a gas chromatography (GC) system (Agilent Technologies, Santa Clara, CA, USA) equipped with a GC column (DB-FFAP 123-3253, 50 m × 0.32 mm × 0.50 *μ*M), flame ionization detector, and autosampler. Nitrogen was used as the carrier gas with a flow rate of 1.4 ml/min a split ratio of 10 : 1. The sample injection volume was 1 *μ*l, and inlet and detector temperatures were 200°C and 240°C, respectively, based on the protocol by Demingne and Remesy [12]. Acetic acid, propionic acid, and butyric acid contents were used as standards.

### 2.7. Histopathological Analysis

Colon tissue obtained from the sacrificed SD rats was fixed with 10% formalin for 24 h. The colon was embedded in paraffin wax and then sectioned into 3 mm thick slices. The slices were stained with hematoxylin and eosin (H&E; Sigma-Aldrich Co, St. Louis, MO, USA). Morphological features of intestinal mucosa cells were observed via light microscopy (ZEISS, Axiovert S100, Germany).

For mucin staining, tissue sections fixed in paraffin (3 *μ*m) were deparaffinized with xylene. After deparaffinization, the tissues were washed with distilled water and stained with Alcian blue for 30 min. Eventually, the morphology of the cryptic cells in the stained colon sections was observed via light microscopy (ZEISS, Axiovert S100, Germany).

### 2.8. Immunohistochemistry Examination

Interstitial cells of Cajal (ICC) are cells distributed in the smooth muscle layer of the intestine that assists in intestinal peristalsis [13]. To observe these cells, paraffin-embedded intestinal tissues were cut into 4 *μ*m sections after staining with H & E. The sections were then deparaffinized using xylene and hydrolyzed for 5 min with different concentrations of ethanol (100%, 90%, 80%, and 70%). Slide glass containing the tissue was heated to 98°C for 20 min for antigen retrieval, followed by preantibody blocking to prevent staining of cells other than ICC. Primary antibody was diluted 1 : 200 using AB c-kit (Santa Cruz; SC-168, Dallas, TX, USA) and reacted at 4°C for 1 day. After the reaction, antibody enhancer was added to the tissue and incubated for 10 min. After 5 min of treatment with chromogen (DAB Substrate 1 ml + DAB chromogen 1-2 drops), the sections were washed in running water for 1-2 min, reacted with hematoxylin, and then washed with PBS and running water, and eventually they were mounted. Stained ICCs were observed under an optical microscope (ZEISS, Axiovert S100, Jena, Germany), and the number of pixels with RGB values was determined using MATLAB.

### 2.9. Statistical Analysis

Data was statistically analyzed using the statistical package for social science (version 12.0). Means and standard deviations were calculated for all measurements. Following one-way analysis of variance (ANOVA), the significance of intergroup differences was confirmed by Tukey's multiple range test at *P* < 0.05.

## 3. Results

### 3.1. Utilization of MOS by Lactic Acid Bacteria *In Vitro*

**Figure 1:**
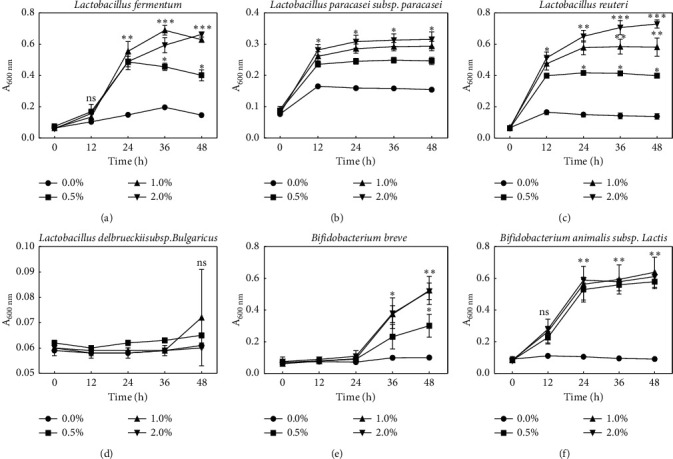
Growth of *Lactobacillus fermentum*, *Lactobacillus paracasei*, *Lactobacillus reuteri*, *Lactobacillus bulgaricus*, *Bifidobacterium breve*, and *Bifidobacterium lactis* in modified PYF broth containing various concentrations of MOS. Bars represent the standard deviation from triplicate determinations. ^*∗*^*P* < 0.05, ^*∗∗*^*P* < 0.01, and ^*∗∗∗*^*P* < 0.001 versus control group (ANOVA followed by *post hoc* Tukey's test). n.s.: not significant.

MOS provided by NEO CREMAR comprised 48.8% of dietary fiber (data not shown). In total, 99% of the total dietary fiber was water-soluble. Polysaccharides are often fermented by intestinal microbes that may selectively use them. Nonprebiotic MOSs reveal different levels of polymerization, and, consequently, the availability of MOS in intestinal microbes may vary. Therefore, the availability of the used MOS for enteric microbes was measured *in vitro* (Figure 1). Figure 1 illustrates the growth of enteric bacteria in medium containing 0%–2% MOS. Lactic acid bacteria except for *L. bulgaricus* showed increased growth in correlation with the amount of MOS added. *L. fermentum* and *Bifidobacterium lactis* started multiplying rapidly 12 h after inoculation into MOS-supplemented medium, whereas *L. paracasei* and *L. reuteri* displayed a rapid increase in cell numbers for 12 h followed by a gradual decrease of the proliferation rate. The growth rate of *B. breve* increased 24 h after treatment commencement, and, in case of *L*. *bulgaricus,* MOS had little effect on the cell growth (Figure 1).

### 3.2. Fecal Pellet Number and Water Content

**Figure 2:**
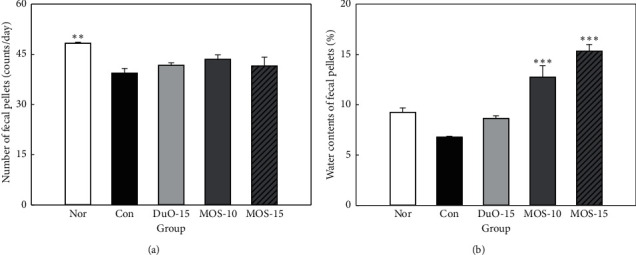
Effect of maltooligosaccharide (MOS) on fecal pellets number (a) and fecal water content (b) in normal and loperamide-induced constipated rats. Nor: normal group, Con: control group, DuO-15: Du-oligo 15%-treated group as positive control, MOS-10: MOS 10%-treated group, and MOS-15: MOS 15%-treated group. The other groups except Nor are loperamide-induced constipation models. Data are represented as the mean ± SE. ^*∗∗*^*P* < 0.01 and ^*∗∗∗*^*P* < 0.001 versus control group (ANOVA followed by *post hoc* Tukey's test).

To evaluate the constipation mitigation effect of MOS, changes in the number of fecal pellets and fecal water content were measured. The number of fecal pellets was 39/day (*n* = 8) in the negative control group (Con), which was significantly lower than that in the normal group (48/day, *n* = 8), as illustrated in Figure 2(a) (*P* < 0.01). The positive control group (DuO-15), as well as the low and high MOS treated groups (MOS-10 and MOS-15) had lower numbers of fecal pellets than those in the normal group, but, within the three groups, the numbers of fecal pellets were similar (42, 44, and 42/day, *n* = 8; Figure 2(a)).

The water content of fecal pellets was the lowest in the negative control group at 6.79%, whereas in the positive control group it was 8.64%, which was similar to 9.26% in the normal group. The fecal water contents of the groups treated with low and high concentrations of MOS (12.74% and 15.34%) were significantly higher than those in the normal group and increased in a concentration-dependent manner (*P* < 0.001; Figure 2(b)).

### 3.3. Intestinal Transit Ratio

**Figure 3:**
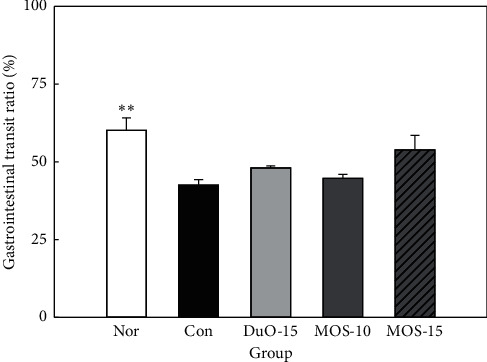
Effect of maltooligosaccharide (MOS) on gastrointestinal transit ratio in normal and loperamide-induced constipated rats. Nor: normal group, Con: control group, DuO-15: Du-oligo 15%-treated group as positive control, MOS-10: MOS 10%-treated group, and MOS-15: MOS 15%-treated group. The other groups except Nor are loperamide-induced constipation models. Data are represented as the mean ± SE. ^*∗∗*^*P* < 0.01 versus control group (ANOVA followed by *post hoc* Tukey's test).

Intestinal transit ratio is an import indicator of constipation [14]. This ratio was measured to compare the effect of MOS on intestinal motility (Figure 3). In the constipated control group without MOS treatment, the intestinal ratio was 42.51% (*n* = 8). Groups subjected to MOS treatment and the positive control group treated with dual-oligosaccharide presented an increased intestinal transit ratio compared to that in the control group. In particular, the group treated with a higher dose of MOS had a transit ratio of 53.92%, which was substantially higher than that of the control group.

### 3.4. SCFA Changes in Cecum

**Figure 4:**
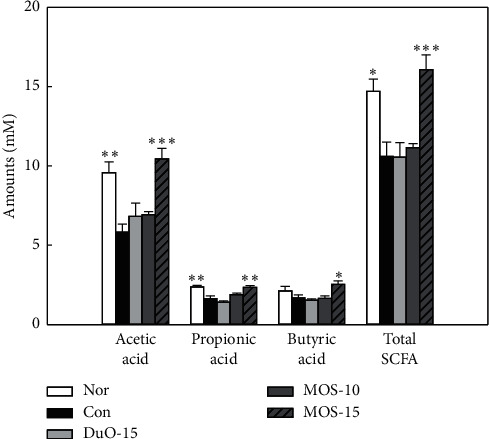
The concentration of acetic acid, butyric acid, propionic acid, and total short-chain fatty acid on cecum in normal and loperamide-induced constipated rats. Nor: normal group, Con: control group, DuO-15: Du-oligo 15%-treated group as positive control, MOS-10: maltooligosaccharide 10%-treated group, and MOS-15: MOS 15%-treated group. The other groups except Nor are loperamide-induced constipation models. Data are represented as the mean ± SE. ^*∗*^*P* < 0.05, ^*∗∗*^*P* < 0.01, and ^*∗∗∗*^*P* < 0.001 versus control group (ANOVA followed by *post hoc* Tukey's test).

SCFAs, such as acetic acid, propionic acid, and butyric acid, are rapidly absorbed by the intestinal mucosa and are used as a major energy source by colonic mucosa [15]. Therefore, to investigate the effect of MOS administration on the production of SCFAs that help promote intestinal health, acetic acid, propionic acid, butyric acid, and total SCFA contents in cecum were analyzed via GC (Figure 4). A high level of SCFA content was found in both normal and high dose MOS groups, with levels significantly different from the control group (*P* < 0.05 and *P* < 0.001, respectively). Among acetic acid, propionic acid, and butyric acid, the amount of acetic acid produced was the highest in all groups. SCFA production increased in correlation to the MOS dose. In the Duo-15 positive control group, an increase in SCFA content was detected in comparison to the control group.

### 3.5. Changes in Histological Structure and Mucin Secretion of the Colon

**Figure 5:**
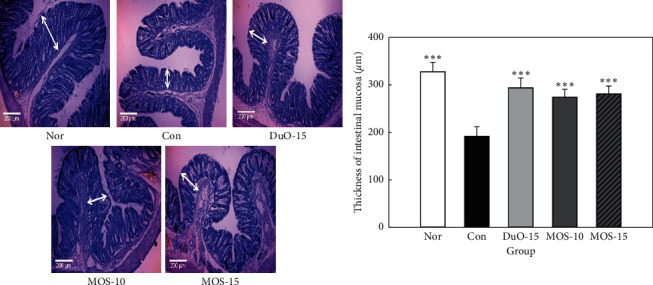
Effect of maltooligosaccharide (MOS) on thickness of intestinal mucosa. Nor: normal group, Con: control group, DuO-15: Du-oligo 15%-treated group as positive control, MOS-10: MOS 10%-treated group, and MOS-15: MOS 15%-treated group. The other groups except Nor are loperamide-induced constipation models. Data are represented as the mean ± SE. ^*∗∗∗*^*P* < 0.001 versus control group (ANOVA followed by *post hoc* Tukey's test).

Figure 5 depicts the effect of MOS intake on mucosal layer thickness in rats with loperamide-induced constipation. The thickness of intestinal mucosa was the lowest in the constipated control group without MOS treatment, which also displayed the lowest intestinal transit ratio among the experimental groups. Both MOS treated groups and the positive control group revealed a higher mucosal layer thickness than that observed in the control group (*P* < 0.001), indicating that intestinal mobility was restored by MOS treatment. In conclusion, mucosal thickness tended to increase in positive correlation with MOS intake.

**Figure 6:**
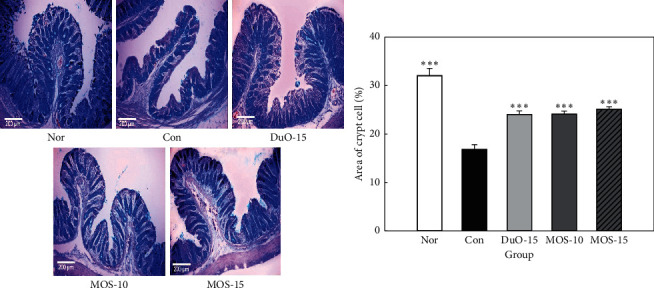
Effect of maltooligosaccharide (MOS) on area of crypt cells. Nor: normal group, Con: control group, DuO-15: Du-oligo 15%-treated group as positive control, MOS-10: MOS 10%-treated group, and MOS-15: MOS 15%-treated group. The other groups except Nor are loperamide-induced constipation models. Data are represented as the mean ± SE. ^*∗∗∗*^*P* < 0.001 versus control group (ANOVA followed by *post hoc* Tukey's test).

Alcian blue is used to stain crypt cells, which are involved in mucin production. As illustrated in Figure 6, the control group treated with loperamide alone presented a reduced level of Alcian blue stained area (presenting mucin) in the mucosal layer of the colon compared to that in the normal group; however, oligosaccharide (Duo-15, MOS-10, and MOS-15) uptake significantly increased the percentage of crypt cells involved in mucin production compared to that in the control group (*P* < 0.001).

### 3.6. Changes in the Area of ICC

**Figure 7:**
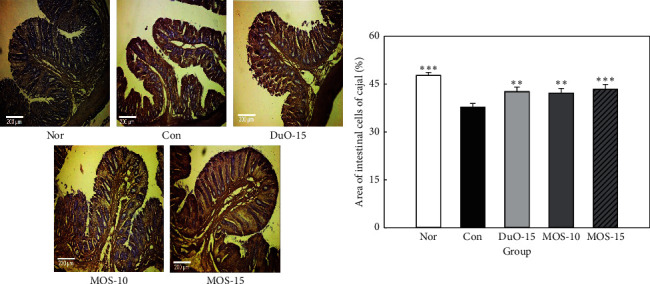
Effect of maltooligosaccharide (MOS) on the area of interstitial cells of cajal (ICC). Nor: normal group, Con: control group, DuO-15: Du-oligo 15%-treated group as positive control, MOS-10: MOS 10%-treated group, and MOS-15: MOS 15%-treated group. The other groups except Nor are loperamide-induced constipation models. Data are represented as the mean ± SE. ^*∗∗*^*P* < 0.01 and ^*∗∗∗*^*P* < 0.001 versus control group (ANOVA followed by *post hoc* Tukey's test).

ICCs regulate the smooth muscles in the intestinal mucosa and play an important role in controlling intestinal motility, including contraction and relaxation of the intestinal muscles [16]. ICC is present in all layers of the colon [17]. In the normal group, the c-kit positive immune response structure is presented in brown (Figure 7).

The constipated group without MOS treatment revealed a significant decrease in the ICC area compared to that in the normal group (*P* < 0.001; Figure 7). Both low dose of MOS and dual-oligosaccharide treatment significantly increased the ICC area in SD rats compared to that in the control rats (*P* < 0.01), and the ICC region significantly increased to the highest level, particularly when the high dose of MOS was administered (*P* < 0.001).

## 4. Discussion

Constipation is a chronic disorder characterized by reduced bowel movements, difficulty in defecation, and incomplete intestinal evacuation sensation [18, 19]. Constipation is often caused by a lack of dietary fiber, insufficient fluid intake, decreased physical activity, drug side effects, hyperthyroidism, and obstruction by colorectal cancer [20]. Altered diet leads to changes in the intestinal microflora, making it safe and sustainable to prevent or relieve constipation. Direct intake of probiotics can induce favorable changes in the intestinal microflora and promote the growth of beneficial bacteria in the body. Another way to improve gut flora is the regular intake of prebiotics such as xylooligosaccharides, GOSs, and FOSs, which are used as nutrients by the gut microbes. Prebiotics improve the intestinal environment by promoting the growth of beneficial bacteria and inhibiting the growth of pathogenic bacteria. MOSs are oligosaccharides produced during the hydrolysis of amylose, a major component in plant starch. In addition to amylase, pullulanase with amylopectin-degrading activity is used for MOS production; however, only amylase was used to manufacture the MOS used in the present study. The typical end products of *α*-amylase activity are branched *α*-limit dextrin and MOSs comprising 2–12 glucose units [21].

Changes in fecal pellet number, weight, and water content in rats with loperamide-induced constipation are important factors that need to be improved while aiming to relieve constipation. Previous studies have reported a marked reduction in the fecal pellet count, weight, and water content, in rats treated with loperamide [22, 23], whereas prebiotic intake is known to improve the fecal parameters [24, 25]. The MOS used in this study also improved the fecal pellet number and water content (Figures 2 and 3).

Compared to the constipated control group, high concentration MOS treatment tended to increase the gastrointestinal transit ratio (Figure 3). The effect of MOS on the intestinal transit ratio is presumably due to the limit dextrin. *α*-amylase produces a mixture of limit *α*-dextrins, short linear oligosaccharides, and glucose during MOS production [26]. Recently, nondigestible dextrin, *α*-cyclodextrin, and dextran were observed to increase intestinal SCFA production, in particular, acetate and propionate, in an *in vitro* fecal fermentation model of human colonic microbiota [27]. Moreover, functional oligosaccharides can inhibit and alleviate intestinal diseases. For example, FOS, MOS, and GOS are known to be suitable substrates for *Bifidobacterium* sp. and *Bacteroides* sp. [28]. The production of SCFAs by these intestinal microorganisms stimulates intestinal peristalsis and increases the humidity of the stool by osmotic pressure [4]. In this study, MOS administration significantly increased the total SCFA content compared to that in the control (*P* < 0.001; Figure 4). Mammalian intestine has a complex microbial ecosystem that is not completely understood. Short-term [29] and long-term [30] dietary carbohydrate intake affects the human fecal microflora. The use of specific starches (e.g., complex resistant starch granules, soluble MOSs, and amylopectin) by intestinal microorganisms depends on the specific activity of the GH13 enzyme and the type of glycan absorption system that works with the enzyme [31].

In Figure 6, goblet cells are stained with Alcian blue in normal rats, and the fine cells in the crypts are well-aligned. This indicates that the goblet cells produce normal mucins containing sulfomucin. Loperamide administration significantly reduced the number of cryptic cells compared to those in the normal group (*P* < 0.001), indicating that it contained less mucin. Higher counts of unstained and stained goblet cells were observed in MOS-supplemented rats compared to those in the loperamide control group, indicating an increase in the goblet cells and intracellular mucin levels resulting in improved mucus secretion [32]. Mucin, a major component of lumen mucus, serves to protect the colonic mucosa from mechanical and chemical damage [33]. Loperamide reduces both synthesis and storage of mucins in cryptic cells [34]. As illustrated in Figure 6, MOS administration improved mucin production, thereby facilitating the passage of feces along the colon and reducing the risk of mucosal damage by decreasing the exposure time of colonic mucosa to potential risk factors.

Compared to the constipated control group without MOS treatment, the ICC count in the MOS treated groups was significantly higher (Figure 7, *P* < 0.01 and *P* < 0.001, respectively). ICC plays a pivotal role in controlling intestinal motility and is present in all layers of the colon [15, 35]. A decrease in ICC leads to slow bowel movements and decreased smooth muscle contractile activity, which can cause constipation. In addition, ICC numbers are markedly reduced during constipation, which can further amplify the constipation [17, 36].

## 5. Conclusions

In this study, MOS increased the water content of the stool, intestinal passage rate, and production of SCFAs, such as acetic acid, propionic acid, and butyric acid, in the intestinal tract. Moreover, it promoted mucus production by the epithelial cells of the small intestine and increased the number of ICC compared to that in the control rats without MOS treatment. MOS alleviated the symptoms of loperamide-induced constipation as it contributed to the stabilization of the intestinal mucosal barrier. Ingestion of MOS can be recommended as an effective alternative for treating constipation.

## Data Availability

The data used to support the findings of this study are included in the article.
